# Starches from Different Tiger Nut Varieties: A Comparative Study of Multi-Scale Structural Characteristics, Functional Properties, and In Vitro Digestibility

**DOI:** 10.3390/foods15111915

**Published:** 2026-05-28

**Authors:** Youkang Chen, Chenghao Liu, Zhuqing Zhao, Runhua Ji, Yang Yang, Xuebo Liu, Min Zhang, Yutang Wang

**Affiliations:** 1 College of Food Science and Engineering, Northwest A & F University, Yangling 712100, China; 15191567418@163.com (Y.C.); 13256525507@163.com (C.L.); 13485402986@163.com (Z.Z.); jirunhua00@163.com (R.J.); xueboliu@nwsuaf.edu.cn (X.L.); 2 College of Food Engineering, Harbin University of Commerce, Harbin 150028, China; foodyangyang@163.com; 3 China Food Flavor and Nutrition Health Innovation Center, Beijing Technology and Business University, Beijing 100048, China

**Keywords:** tiger nut starch, resistant starch, structure–property relationship, in vitro digestion

## Abstract

Tiger nuts are rich in various nutrients, with starch being a key component that holds potential for applications in foods with lower digestibility. However, the varietal dependence of their functional properties has not yet been comprehensively characterized. In this study, starches from different tiger nut varieties—Yunnan Large-seeded Tiger Nut (YLL), Yunnan Small-seeded Tiger Nut (YSL), and Zhongyousha No.1 (ZYS)—were systematically analyzed to investigate differences in their multi-scale structures, functional properties, and digestibility. The results revealed significant varietal differences in amylose content (22.82–26.99%), granule size (D_50_ 8.66–10.23 μm), and short-range molecular order. All starches exhibited A-type crystalline structures, though their relative crystallinity (25.40–30.60%) differed significantly. In vitro digestion profiles demonstrated a two-phase hydrolysis pattern across all varieties, a rapid digestion phase (0–30 min) followed by a slow digestion phase (30–120 min), with resistant starch content ranging from 33.55% to 38.31%. Among the three varieties, higher amylose content and crystallinity were generally associated with enhanced digestive resistance and lower peak viscosity, while gelatinization temperature appeared to be more closely related to granule size than to crystallinity. Peak gelatinization temperature (*T*_P_) ranged from 67.40 to 68.16 °C and peak viscosity (PV) from 7030.0 to 7749.5 mPa·s, with YSL, which had a relatively broad granule size distribution and the lowest crystallinity, exhibiting the highest *T*_P_ and PV. This study provides a reference for understanding the structure–property relationships of tiger nut starches across different varieties and their potential application in functional foods.

## 1. Introduction

Starch ranks among the most abundant carbohydrates in the human diet and is extensively employed across both food and industrial sectors owing to its functional versatility. Nevertheless, the rapid enzymatic digestibility of conventional starches frequently induces pronounced postprandial glycemic fluctuations—a phenomenon intimately linked to the pathogenesis of metabolic disorders such as diabetes and obesity. Accordingly, the development of low-glycemic-index (GI) foods capable of moderating starch digestion and improving glycemic homeostasis has emerged as a research priority within the food and nutritional sciences [1,2]. Resistant starch (RS), defined as the starch fraction that escapes enzymatic hydrolysis in the small intestine, has attracted considerable attention for its well-documented capacity to modulate postprandial glycemic responses and promote metabolic health [3]. RS is typically classified into five types: RS1 (physically inaccessible starch), RS2 (native granular starch with resistance arising from its compact crystalline structure), RS3 (retrograded starch formed upon cooling after gelatinization), RS4 (chemically modified starch), and RS5 (amylose–lipid complexes) [4]. Among these, RS2 and RS3 are the most directly relevant to starch-based food systems, as their formation and stability are governed by the multi-scale structural features of the starch granule. Native starch granules with dense crystalline organization and compact granule architecture are intrinsically resistant to enzymatic attack, as the tightly packed molecular arrangement limits enzyme diffusion and binding to the granule interior [5].

Tiger nut (*Cyperus esculentus* L.), a perennial tuberous plant of the Cyperaceae family native to the Mediterranean coastal regions of Africa, has undergone substantial expansion in cultivation across China in recent years [6]. This crop exhibits a favorable nutritional profile. Starch constitutes approximately 14–37% of its dry weight, complemented by appreciable levels of lipids (25–35%), dietary fiber, protein, bioactive constituents such as phenolics and sterols, and minerals, which collectively confer antioxidant, hypolipidemic, and cardioprotective benefits [7,8,9]. Moreover, tiger nut demonstrates considerable tolerance to abiotic stresses and thrives in marginal and saline-alkali soils, thereby representing an environmentally sustainable starch source. Prior investigations have established that tiger nut starch is characterized by high amylopectin content, an A-type crystalline polymorph, excellent freeze–thaw stability, and substantial gel strength. Notably, this starch also exhibits a comparatively retarded enzymatic digestion rate and elevated resistant starch content, underscoring its promising potential for incorporation into foods with reduced digestibility [10,11,12].

Notwithstanding the considerable promise of tiger nut in food processing and nutritional health, extant research has largely centered on oil extraction and the development of plant-based beverages, whereas systematic characterization of its starch component remains relatively sparse [13,14]. Although preliminary work has documented inter-varietal differences in tiger nut starch properties [15], these studies have typically focused on either structural characterization or the measurement of functional behavior, without integrating multiscale structural analysis (spanning granule morphology, long-range crystallinity, and short-range molecular order) with comprehensive functional evaluation (including rheological properties, thermal stability, and biphasic digestion kinetics) within a single analytical framework [15]. Consequently, the relationships between structural features at different organizational scales and their combined influence on the functional and digestive properties of tiger nut starch remain poorly understood.

It is now well recognized that variety-dependent differences in starch structural features are closely associated with both functional behavior and digestive properties. Key structural attributes—including granule morphology, crystalline polymorph, amylose-to-amylopectin ratio, and chain-length distribution—have been shown to influence gelatinization behavior, thermal characteristics, rheological performance, and in vitro enzymatic susceptibility, thereby affecting the suitability of starch for targeted food applications [14,15,16]. For instance, investigations on cereal and tuber crops have consistently demonstrated that elevated amylose content and a greater proportion of long amylopectin chains are positively correlated with higher gelatinization temperatures, enhanced gel rigidity, and reduced enzymatic digestibility [17,18]. Investigating the relationships between the multi-scale structural features of tiger nut starch and its functional properties is, therefore, of considerable significance for delineating its potential application domains. Furthermore, given the ongoing expansion of tiger nut cultivation and the increasing diversification of varietal resources in China, a systematic understanding of inter-varietal differences in starch quality holds substantial practical relevance for optimizing processing strategies and guiding the development of specialized functional foods.

Against this backdrop, we hypothesized that the multi-scale structural features of tiger nut starch—particularly amylose content, granule size distribution, long-range crystallinity, and short-range molecular order—differ among varieties, and that these differences are associated with variation in functional properties and in vitro digestibility. To test this hypothesis, starch was isolated from the tubers of three major tiger nut varieties cultivated in China (detailed varietal information and provenance are provided in Section 2.1). A systematic comparative analysis was conducted to characterize the multi-scale structural features of the isolated starches, encompassing granule morphology, crystalline organization, and molecular order. Key physicochemical and functional attributes—including pasting behavior, thermal properties, and freeze–thaw stability—were comprehensively evaluated, and in vitro digestibility was assessed via simulated enzymatic digestion.

## 2. Materials and Methods

### 2.1. Materials

**Figure 1:**
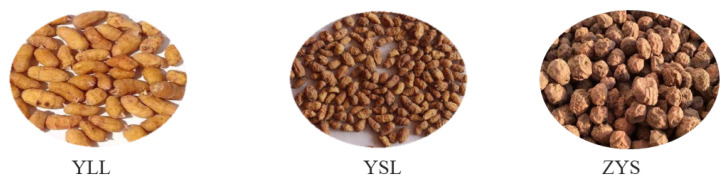
Appearance of the three tiger nut varieties.

Three representative tiger nut varieties from China were selected based on their morphological characteristics. Their main features are presented in Table 1 and Figure 1.

The enzymes porcine pancreatic α-amylase and *Aspergillus niger amyloglucosidase* were purchased from OriLeaf Bio-Technology Co., Ltd. (Shanghai, China), and all other chemicals were analytical grade unless stated otherwise.

### 2.2. Sample Preparation and Starch Extraction

Tiger nut tubers were cleaned to remove impurities and homogenized with distilled water at a 1:15 (*w*/*v*) ratio using a high-speed blender. The slurry was sequentially screened through 60-, 100-, and 200-mesh sieves. After refrigeration at 4 °C for 12 h, the supernatant was discarded. The resulting precipitate was washed three times with distilled water, followed by a final wash with anhydrous ethanol, then dried at 50 °C for 48 h, and finally ground and passed through a 200-mesh sieve to obtain the native starch [19]. Determined in Section 2.3.1, the starch purity of the three varieties was approximately 93% (dry basis).

### 2.3. Multi-Scale Structural Characteristics

#### 2.3.1. Determination of Starch and Apparent Amylose Content

The total starch content, used as an indicator of starch purity, was determined using an acid hydrolysis method to convert the starch into glucose, followed by quantification of reducing sugars via the dinitrosalicylic acid (DNS) assay. The final starch content was calculated using a conversion factor of 0.9. Prior to the acid hydrolysis step, the starch samples were washed alternately with petroleum ether and anhydrous ethanol (three times each). Each wash was conducted with continuous shaking for 1 h, and the sample was recovered by centrifugation at 5000 rpm for 10 min. This pre-treatment was performed to remove residual lipids, free sugars, and other potentially interfering non-starch components.

Apparent amylose content was measured according to an established iodine-binding protocol [10]. A 50 mg starch sample was dissolved in 10 mL of 1 mol/L NaOH by incubating it in a boiling water bath for 30 min. Following cooling, the volume was made up to 50 mL. Subsequently, 2.5 mL of this solution was mixed with acetic acid and an I_2_-KI reagent. After a 20-min reaction period, the absorbance was read at 620 nm. Calculations were based on a corn amylose standard curve.

#### 2.3.2. Particle Size Distribution

The particle size distribution was determined using a laser particle size analyzer (LS13320, Beckman Coulter, Inc., Brea, CA,USA) equipped with a Universal Liquid Module (ULM). Starch samples were dispersed in distilled water to form a suspension (0.1% *w*/*v*). Measurements were performed in triplicate. The particle size distribution curves were recorded, and parameters including D_10_, D_50_, D_90_, and volume-weighted mean diameter (D_m_) were obtained.

#### 2.3.3. Scanning Electron Microscopy

Dried starch granules were uniformly distributed onto the surface of a conductive double-sided tape. To enhance conductivity, the samples were sputter-coated with a thin layer of gold/palladium for 5 min using a sputter coater. The granule morphology and surface features were then examined using a scanning electron microscope (SEM; Model Nano SEM-450, Thermo Fisher Scientific, Waltham, MA, USA) operated at an accelerating voltage of 5–10 kV.

#### 2.3.4. Long-Range Crystalline Structure

X-ray diffraction (XRD) analysis of the 200-mesh starch samples was performed using a diffractometer (D8 Advance A25, Bruker Ltd., Karlsruhe, Germany) with Cu-Kα radiation (40 kV, 30 mA). Scans were collected over a 2θ range of 5–50° at a scanning speed of 4°/min and a step size of 0.02°. Baseline correction of the XRD data was carried out using Jade 6.5 software. Relative crystallinity (RC) was calculated from the diffraction patterns by integrating the areas of the crystalline peaks (*Ac*) and the amorphous background (*Aa*) according to Equation (1) [19]:

(1) $$\mathrm{RC}=\frac{Ac}{Ac+Aa}\times 100\%$$

#### 2.3.5. Short-Range Crystalline Structure

Fourier-transform infrared (FT-IR) spectroscopy was employed to characterize the short-range molecular order of the starch samples using a spectrometer (TENSOR 27, Bruker Ltd., Karlsruhe, Germany). Starch samples (2 mg) were thoroughly mixed with dried KBr (200 mg) and pressed into transparent pellets. Spectra were recorded over the range of 400–4000 cm^−1^ at a resolution of 4 cm^−1^, with 32 scans co-added, against a pure KBr background. All spectra were baseline-corrected, smoothed, and normalized using Omnic 8.0 software. The characteristic absorption bands in the 800–1200 cm^−1^ region were subjected to deconvolution, with a half-peak width of 25 cm^−1^ and an enhancement factor of 2.0 [19]. The intensity ratios R_1047/1022_ and R_1022/995_ were calculated from the deconvoluted spectra using peak heights.

### 2.4. Physicochemical Properties

#### 2.4.1. Swelling Power (SP) and Solubility (SOL)

All measurements were performed in triplicate. A starch sample (50.0 mg, dry basis, *m*_1_) was suspended in 5 mL of distilled water and heated in a water bath at 55, 65, 75, 85, and 95 °C for 30 min each. After cooling to ambient temperature, centrifugation was performed at 4000 rpm for 15 min. The supernatant was carefully decanted into a pre-weighed aluminum box and dried at 110 °C to constant weight (*m*_2_). The wet precipitate was weighed (*m*_3_). Swelling power (SP) and solubility (SOL) were calculated using Equations (2) and (3), respectively [20]:

(2) $$\mathrm{SOL}=\frac{m_{2}}{m_{1}}\times 100\%$$

(3) $$\mathrm{SP}=\frac{m_{3}}{m_{1}-m_{2}}\times 100\%$$

#### 2.4.2. Water (WAC) and Oil (OAC) Absorption Capacity

All measurements were performed in triplicate. A starch sample (0.50 g, *m*_1_) was mixed with 5 mL of deionized water or edible soybean oil and incubated at 30 °C for 30 min with intermittent shaking. The mixture was then centrifuged at 4000 rpm for 15 min. The supernatant was discarded, and the sediment was weighed (*m*_2_ for water, *m*_3_ for oil). WAC and OAC were calculated as follows [21]:

(4) $$\mathrm{WAC}\;(g/g)=\frac{m_{2}-m_{1}}{m_{1}}$$

(5) $$\mathrm{OAC}\;(g/g)=\frac{m_{3}-m_{1}}{m_{1}}$$

#### 2.4.3. Paste Clarity

A starch suspension (1%, *w*/*v*) was prepared in deionized water and gelatinized by heating in a boiling water bath with constant magnetic stirring for 30 min, followed by immediate cooling to room temperature. The gelatinized sample was stored sealed at 4 °C for 96 h. Measurements were taken after 0, 24, 48, 72, and 96 h of storage. Before each measurement, the samples were equilibrated to room temperature and vortexed thoroughly. The transmittance was measured at 640 nm using a UV-Vis spectrophotometer (UV-1900i, Shimadzu Instruments (Suzhou) Co., Ltd., Suzhou, China) against a deionized water blank. All measurements were performed in triplicate [22].

#### 2.4.4. Pasting Properties

Pasting properties were determined using a Rapid Visco Analyser (RVA 4500, Perten Instruments, Hägersten, Sweden). A starch suspension (3.0 g, dry basis, in 25.0 g distilled water) was prepared and subjected to the following temperature profile: equilibration at 50 °C for 2 min, heating to 95 °C over 4.5 min, holding at 95 °C for 3 min, cooling to 50 °C over 2.5 min, and holding at 50 °C for 2 min. Pasting curves were recorded, and key parameters—peak viscosity, trough viscosity, breakdown viscosity, final viscosity, and setback viscosity—were analyzed. All measurements were performed in triplicate [22].

#### 2.4.5. Rheological Properties

The rheological properties were measured using a discovery hybrid rheometer (DHR-1, TA Instruments, New Castle, DE, USA) with a 40 mm parallel plate geometry and a 0.05 mm gap. A 6% (*w*/*w*) starch suspension was gelatinized in a boiling water bath for 30 min, cooled, and loaded onto the Peltier plate. After loading, a thin layer of silicone oil was applied around the periphery of the sample to prevent moisture evaporation during measurement. The sample was equilibrated at 25 °C for 120 s before testing. Steady shear tests were conducted by ramping the shear rate from 0.1 to 100 s^−1^. The resulting flow curves were fitted to the Ostwald–de Waele model to obtain the consistency coefficient (*K*) and flow behavior index (*n*).

(6) $$\tau =K\times r^{n}$$

For dynamic rheology, the linear viscoelastic range (LVR) was first identified via amplitude sweep testing (0.1–10% strain, 1.0 rad/s). Based on this, a 2% fixed strain was selected. The paste was then analyzed at 25 °C under 2% strain over 0.1–100 rad/s, recording variations in elastic modulus (G′), viscous modulus (G″), and loss tangent (tan δ at 10 s^−1^) [19].

#### 2.4.6. Thermal Properties

The thermal properties of the starch were analyzed using a differential scanning calorimeter (DSC, Q2000, TA Instruments, New Castle, DE, USA). Starch samples (3.0 mg, dry basis) were accurately weighed into aluminum pans, mixed with 9 μL of distilled water, and hermetically sealed. The sealed pans were then equilibrated at 4 °C for 12 h prior to analysis. The analysis involved heating the samples from 30 to 120 °C at a scanning rate of 10 °C/min under a nitrogen purge. An empty sealed pan was used as a reference. The onset (*To*), peak (*Tp*), and conclusion (*Tc*) temperatures, as well as the gelatinization enthalpy (Δ*H*), were determined from the endothermic curves [19].

#### 2.4.7. Freeze–Thaw Stability

The freeze–thaw stability was evaluated in triplicate. A starch paste (6%, *w*/*w*) was gelatinized in a boiling water bath for 30 min and cooled to room temperature. Aliquots of the paste were subjected to repeated freeze–thaw cycles, each consisting of freezing at −20 °C for 22 h followed by thawing at 30 °C for 2 h, for up to 5 days. After each cycle, samples were centrifuged at 3000 rpm for 20 min, and the supernatant was carefully removed. Syneresis was calculated using Equation (7):

(7) $$\mathrm{Sys}=\frac{m_{2}-m_{3}}{m_{2}-m_{1}}\times 100\%$$

where *m*_1_ is the weight of the empty centrifuge tube, *m*_2_ is the total weight of the tube and paste before freezing, and *m*_3_ is the total weight of the tube and precipitate after centrifugation.

### 2.5. In Vitro Digestion Simulation

In vitro starch digestibility was determined according to the method of Zhu et al. [23] with slight modifications. Briefly, starch samples (5 mg/mL in 0.5 M sodium acetate buffer, pH 5.2) were equilibrated at 37 °C for 10 min. The enzymatic reaction was initiated by adding pancreatin (100 U of α-amylase activity/mL) and amyloglucosidase (260 U/mL). Aliquots (200 μL) were withdrawn at 0, 10, 20, 30, 60, 90, 120, and 180 min, immediately inactivated in a boiling water bath for 5 min, and centrifuged (10,000 rpm, 15 min). The reducing sugar content in the supernatant was measured using the DNS method. Rapidly digestible starch (RDS), slowly digestible starch (SDS), and resistant starch (RS) contents were calculated as follows:

(8) $$\mathrm{RDS}=\frac{(G_{20}-G_{0})\times 0.9}{W}\times 100\%$$

(9) $$\mathrm{SDS}=\frac{(G_{120}-G_{20})\times 0.9}{W}\times 100\%$$

(10) RS=1−RDS−SDS

where *W* is the total starch content (mg), *G*_0_ is free glucose content (mg), and *G*_20_ and *G*_120_ are glucose contents at 20 and 120 min (mg), respectively.

To characterize the detail digestion rates of the samples, the correlation between the digested starch fraction and digestion time was analyzed using the 1st-order digestion equation as follows [19]:

(11) $$C_{t}=C_{\infty }\times (1-e^{-k}t)$$

where *C_t_* and *C_∞_* were the fractions of the digested starch at the digestion time *t* and termination, respectively; and *k* was the digestion rate constant.

To obtain the *k* value, the above equation was transformed into a logarithm of slope (LOS) type as follows [19]. The 180-min hydrolysis value was selected as the equilibrium hydrolysis extent (C∞) for LOS model fitting. This selection was based on the observation that the hydrolysis curves approached a plateau between 120 and 180 min for all samples, with the incremental hydrolysis between 120 and 180 min representing less than 3% of the total hydrolyzed fraction. Using the 180-min time point as C∞ is consistent with established practice in starch digestion kinetics studies, where the hydrolysis extent at the final sampling time point is commonly used as the equilibrium value when the hydrolysis curve has visibly plateaued [19].

(12) $$\ln(\frac{dC_{t}}{dt})=-kt+\ln(C_{\infty }k)$$

In the case that the starch digestion was a single-phase process, the LOS plot was described as a linear one. In the case that the digestion was a multiple-phase process, the piecewise correlations were applied follows [19]:

(13) $$C_{t}=\left\{\begin{array}{ll} C_{1}+C_{1\infty }\left(1-e^{-k_{1}t}\right), & 0\leq t\leq t_{1}\\ C_{2}+C_{2\infty }\left(1-e^{-k_{2}t}\right), & t_{1}\leq t\leq t_{2}\\ \vdots & \\ C_{n}+C_{n\infty }\left(1-e^{-k_{n}t}\right), & t_{n-1}\leq t\leq t_{n} \end{array}\right.$$

where *n* was the phase order; *C_n_* and *C_n∞_* were the fractions of digested starch at the beginning and termination of each phase, respectively; *k*_n_ was the digestion rate constant of each phase; and *t*_n_ was the terminal timepoint of each phase. The transition time between Phase I and Phase II was identified as the point at which the LOS plot displayed a distinct change in slope, consistent with the sequential digestion pattern described for starch containing multiple digestible fractions in the LOS model [24]. For all samples analyzed in this study, a distinct biphasic pattern was observed, with the inflection point consistently occurring at approximately 30 min.

### 2.6. Statistical Analysis

All experiments were performed in triplicate using independently extracted starch samples, and data are presented as mean ± standard deviation (SD). Data processing and tabulation were performed using Excel 2019. For comparisons among the three varieties, one-way analysis of variance (ANOVA) followed by Duncan’s multiple range test was applied using SPSS 22.0. Statistical significance was defined at *p* < 0.05 (*), *p* < 0.01 (**), and *p* < 0.001 (***). All figures were generated using OriginPro 2024.

## 3. Results and Discussion

### 3.1. Multi-Scale Structural Characteristics

#### 3.1.1. Apparent Amylose Content

**Figure 2:**
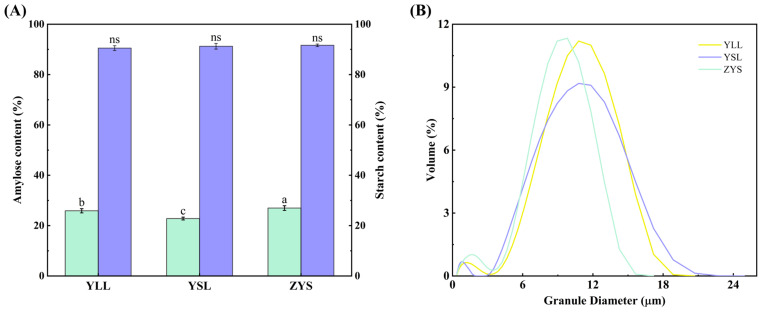
Starch content, amylose content, and granule size distribution of tiger nut starch. (**A**) starch and amylose content, (**B**) granule size distribution. Sample abbreviations: YLL, Yunnan Large-seeded Tiger Nut; YSL, Yunnan Small-seeded Tiger Nut; ZYS, Zhongyousha No.1. Values are presented as mean ± SD (*n* = 3). Different lowercase letters within the same group indicate significant differences. ns, not significant (one-way ANOVA followed by Duncan’s multiple range test, *p* < 0.05).

The amylose-to-amylopectin ratio, a key factor influencing starch functionality, varies substantially among cultivars [25]. The apparent amylose contents of the three tiger nut starches are presented in Figure 2A. Statistically significant differences were observed among the varieties (*p* < 0.05): ZYS exhibited the highest amylose content (26.99%), followed by YLL (25.91%) and YSL (22.82%). These results indicate distinct varietal specificity in the starch composition of tiger nuts. Previous investigations have demonstrated that the amylose content of tiger nut starch is influenced by multiple factors, including genotype, growth environment, and agronomic practices [26]. Reported values for native tiger nut starch range from 9.71% to 27.01%, further underscoring the contribution of varietal and cultivation conditions in determining starch composition [27,28].

Amylose is known to enhance gel strength, retrogradation resistance, and film-forming capacity, and is closely associated with starch solubility and resistance to enzymatic digestion. In contrast, amylopectin contributes to viscosity, flow behavior, and freeze–thaw stability during gelatinization [29,30]. Consequently, differences in the amylose-to-amylopectin ratio among tiger nut varieties may influence their suitability for different food applications. Owing to their higher apparent amylose content, starches from ZYS and YLL tended to exhibit greater resistance to digestion and may be considered as potential candidates for foods targeting reduced digestibility. The gel strength and film-forming ability associated with high-amylose starches may offer additional functional benefits in certain food systems [31]. Conversely, the YSL variety, characterized by a higher proportion of amylopectin, displayed higher viscosity development and freeze–thaw stability upon gelatinization. These contrasting functional profiles between varieties suggest that the amylose-to-amylopectin ratio may be a useful indicator for anticipating the processing behavior of tiger nut starches.

#### 3.1.2. Particle Size Distribution

Granule size distribution, a key factor influencing starch functionality [32,33], varied markedly among the three tiger nut varieties (Figure 2B). All samples exhibited a multimodal distribution pattern, comprising a major peak and a secondary peak, both located below 20 μm. The particle volume distribution ranged from 0.38–18.86 μm for YLL, 0.38–22.73 μm for YSL, and 0.38–15.65 μm for ZYS, indicating substantial differences in distribution width among the varieties. The maximum peak percentages were 14.1% for YLL, 12.6% for YSL, and 15.3% for ZYS, suggesting that starch granule size is affected by both cultivar and growth conditions.

Table 2 presents the significant differences (*p* < 0.05) observed in mean diameter (D_m_), D_10_, D_50_, and D_90_ values across varieties. Following the classification scheme proposed by Ren et al. [34], starch granules can be categorized as large (>25 μm), medium (10–25 μm), small (5–10 μm), or very small (<5 μm). According to this framework, ZYS and YSL starches were predominantly composed of small granules, whereas YLL starch consisted mainly of medium-sized granules, consistent with the 0.182–18.53 μm range reported by Wu et al. [27]. This variation in granule size was accompanied by differences in functional performance among the varieties.

#### 3.1.3. Starch Granule Morphology

**Figure 3:**
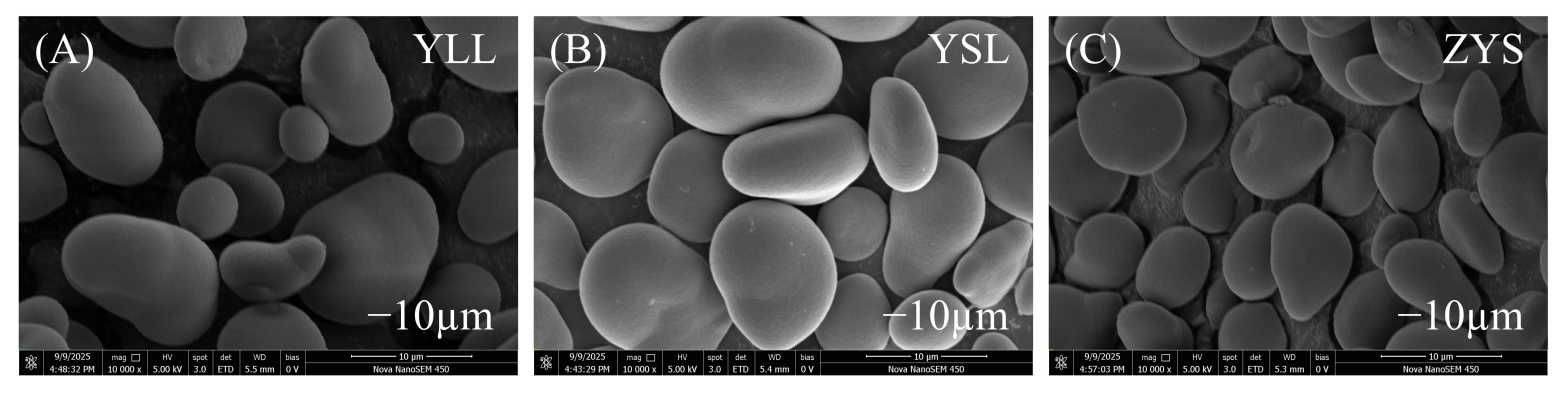
The SEM micrographs of tiger nut starch. (**A**) YLL, (**B**) YSL, (**C**) ZYS. Sample abbreviations: YLL, Yunnan Large-seeded Tiger Nut; YSL, Yunnan Small-seeded Tiger Nut; ZYS, Zhongyousha No.1.

Scanning electron microscopy (SEM) images of the three tiger nut starches are presented in Figure 3. Overall, the starch granules from different varieties exhibited general similarities in morphology; however, discernible differences in granule size distribution were evident through scale bar comparisons, which is consistent with the particle size analysis results. The surfaces of all three starch granules were relatively smooth, and the granules displayed diverse shapes, including nearly spherical, hemispherical, ellipsoidal, and irregular polygonal structures, in agreement with the previously reported morphological characteristics of tiger nut starch, namely smooth surfaces and irregular polygonal granules [15,35].

These morphological distinctions, particularly the variations in granule size and shape, may offer structural insights into the differences in functional properties observed among the starches. The relatively smaller and more uniform granules observed in the ZYS variety were noted alongside its lower peak viscosity and higher dynamic modulus. From a physicochemical standpoint, the larger specific surface area of smaller granules may modulate water absorption kinetics, swelling behavior, and the subsequent development of the gel network during pasting and gelation [36,37], which could contribute to the observed differences in functional behavior. Taken together, granule morphology appears to be one of the factors associated with the variation in functional behavior among YLL, YSL, and ZYS starches among these three varieties.

#### 3.1.4. Long-Range Crystalline Structure

**Figure 4:**
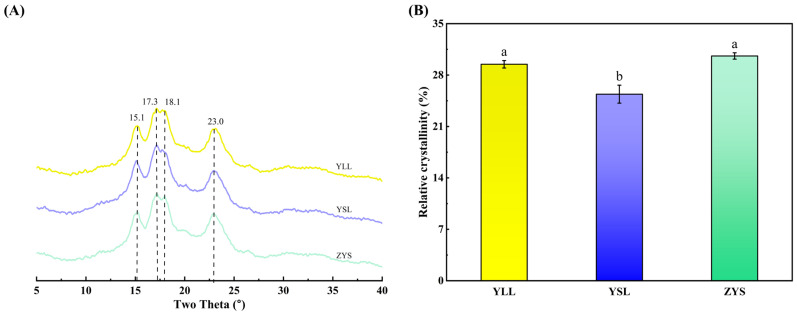
Long-range crystalline structure of tiger nut starch. (**A**) X-ray diffraction (XRD) patterns, (**B**) Relative crystallinity. Sample abbreviations: YLL, Yunnan Large-seeded Tiger Nut; YSL, Yunnan Small-seeded Tiger Nut; ZYS, Zhongyousha No.1. Values are presented as mean ± SD (*n* = 3). Different lowercase letters within the same group indicate significant differences (one-way ANOVA followed by Duncan’s multiple range test, *p* < 0.05).

Starch crystallinity arises from the tight packing of amylopectin double helices into ordered domains [19]. The X-ray diffraction patterns of all three tiger nut starches exhibited clear peaks at 15.1°, 17.3°, 18.1°, and 23° (Figure 4A), indicative of an A-type crystalline polymorph. From a crystallographic perspective, A-type starches contain an orthorhombic or monoclinic unit cell, whereas B-type starches adopt a hexagonal unit cell; C-type starches represent a superposition of A- and B-type polymorphs [38]. The A-type polymorph assignment is consistent with previous reports [39,40]. In contrast, a C-type diffraction pattern has also been documented for tiger nut starch [12]. This discrepancy could be attributed to factors such as varietal differences, growth environment, amylose-to-amylopectin ratio, and crystal size [12,41].

Figure 4B presents the relative crystallinity values of the three starches, 29.47% (YLL), 25.40% (YSL), and 30.60% (ZYS), with significant differences among them (*p* < 0.05). The relationship between crystallinity and amylose content was not strictly linear [42]. The differences in crystallinity may help to understand the variation in functional behaviors observed among the varieties. Specifically, the higher crystallinity of ZYS was noted alongside its higher pasting temperature and reduced digestion rate. These observations are consistent with the expectation that ordered crystalline regions offer greater resistance to swelling and enzymatic attack [43,44]. Conversely, the lower crystallinity of YSL was accompanied by differences in swelling power and pasting viscosity profile.

#### 3.1.5. Short-Range Crystalline Structure

**Figure 5:**
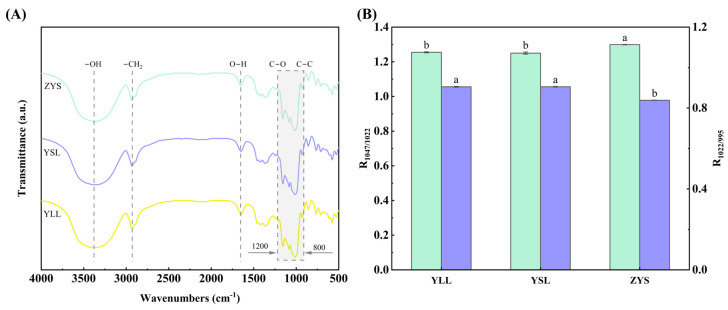
Short-range crystalline structure of tiger nut starch. (**A**) Fourier-transform infrared (FTIR) spectra. (**B**) Intensity ratios R_1047/1022_ and R_1022/995_. Sample abbreviations: YLL, Yunnan Large-seeded Tiger Nut; YSL, Yunnan Small-seeded Tiger Nut; ZYS, Zhongyousha No.1. Values are presented as mean ± SD (*n* = 3). Different lowercase letters within the same group indicate significant differences (one-way ANOVA followed by Duncan’s multiple range test, *p* < 0.05).

FTIR spectroscopy was employed to probe the short-range ordered molecular structure of the three tiger nut starches. As shown in Figure 5A, their FTIR spectra displayed similar profiles, with characteristic absorption bands observed in the 4000–400 cm^−1^ region corresponding to vibrational modes of fundamental functional groups. Key absorption bands were observed at O–H stretching vibrations (3000–3600 cm^−1^) [45], intramolecular hydrogen bonding (≈1000 cm^−1^), CH_2_ stretching (≈2930 cm^−1^), and O–H bending (≈1650 cm^−1^) [20]. The 800–1300 cm^−1^ fingerprint region, which is sensitive to conformational changes, arises from coupled C–O and C–C vibrations [46].

The relationship between the R_1047/1022_ ratio and starch ordered structure was originally established in potato starch (B-type) systems [47], and has subsequently been applied to starches of different crystalline polymorphs [48,49]. The absorption bands at 1047 cm^−1^ and 1022 cm^−1^ are indicative of the crystalline and amorphous domains in starch, respectively. The intensity ratio R_1047/1022_ serves as a measure of short-range structural order, with higher values reflecting a greater degree of ordered molecular arrangement [42]. Additionally, the ratio R_1022/995_ is associated with the relative proportion of amorphous to hydrated ordered domains; a lower value typically indicates a more ordered molecular structure [42]. As presented in Figure 5B, ZYS exhibited significantly higher R_1047/1022_ and lower R_1022/995_ values compared with YLL and YSL (*p* < 0.05). Both ratios consistently indicate that ZYS possessed a more ordered short-range molecular arrangement, with stronger intramolecular hydrogen bonding and greater double-helix stability. These results indicate that short-range structural order in tiger nut starch varies among the varieties examined, in agreement with findings reported for loquat and rice starches [42,50].

### 3.2. Physicochemical Properties

#### 3.2.1. Swelling Power and Solubility

**Figure 6:**
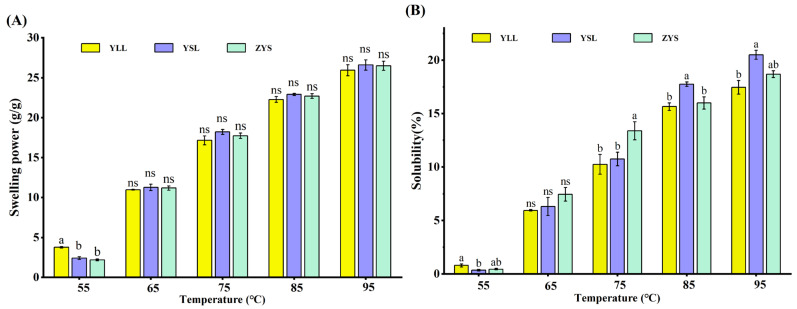
The swelling power and solubility of tiger nut starch. (**A**) swelling power, (**B**) solubility. Sample abbreviations: YLL, Yunnan Large-seeded Tiger Nut; YSL, Yunnan Small-seeded Tiger Nut; ZYS, Zhongyousha No.1. Values are presented as mean ± SD (*n* = 3). Different lowercase letters within the same group indicate significant differences. ns, not significant (one-way ANOVA followed by Duncan’s multiple range test, *p* < 0.05).

Swelling power (SP) reflects the capacity of starch granules to absorb water and expand upon heating, whereas solubility (SOL) indicates the extent of soluble material leaching from the granules during gelatinization [41]. The SP and SOL profiles of the three tiger nut starches measured at temperatures ranging from 55 to 95 °C are presented in Figure 6A,B. Significant swelling occurred in all three starches only above 65 °C, with SP increasing markedly as the temperature increased. At 75 °C, SP rose sharply and then plateaued between 85 and 95 °C. The maximum SP of all three starches was reached at 95 °C. Overall, YSL exhibited the highest SP, whereas ZYS displayed the lowest. Previous studies suggest that amylose restricts granule hydration and swelling, whereas smaller starch granules tend to possess a higher affinity for water [51]. ZYS—which had the highest amylose content and the smallest granule size—showed the lowest SP, suggesting that the restrictive effect of its high amylose content outweighed the swelling-promoting effect associated with its small granule size. The dominant role of amylose in limiting swelling is further supported by the behavior of YSL: this variety had the lowest amylose content and the largest granules, yet exhibited the highest SP. This indicates that, among the structural factors examined, the amylose-to-amylopectin ratio appeared to be the primary determinant of swelling capacity in these three varieties. These results indicate that SP is influenced by multiple structural factors [20,34]; in this study, the amylose-to-amylopectin ratio appeared to show a stronger association with SP variation than granule size. Furthermore, among the three varieties, those with higher relative crystallinity generally displayed lower SP, which is consistent with the expectation that crystalline compactness restricts water penetration into granules.

The solubility of the three starches remained relatively low below 65 °C but increased sharply above this temperature, with significant differences emerging after 85 °C (Figure 6B). At 95 °C, SOL ranged from 17.45% (YLL) to 20.50% (YSL). This indicates that both SP and SOL were influenced by variety and temperature [20]. As temperature increases, the hydrogen bonds stabilizing starch double helices dissociate and are replaced by water–starch hydrogen bonds, thereby facilitating amylose leaching and enhancing solubility [52]. Among the three varieties, an inverse trend was noted between amylose content and SOL; specifically, YSL, with the lowest amylose content, exhibited the highest SOL, whereas ZYS, with the highest amylose content, showed the lowest SOL, suggesting that amylose may restrict solubilization to some extent. These differing SP and SOL profiles suggest that the three varieties may respond differently during thermal processing.

#### 3.2.2. Water and Oil Absorption Capacity

Water absorption capacity (WAC) characterizes the ability of starch granules to bind and retain water, whereas oil absorption capacity (OAC) reflects the capacity of dried starch to interact with lipids via capillary forces and non-covalent interactions [41]. Both WAC and OAC have been reported to be influenced by structural attributes such as the amylose-to-amylopectin ratio, granule size, and crystallinity [21].

**Figure 7:**
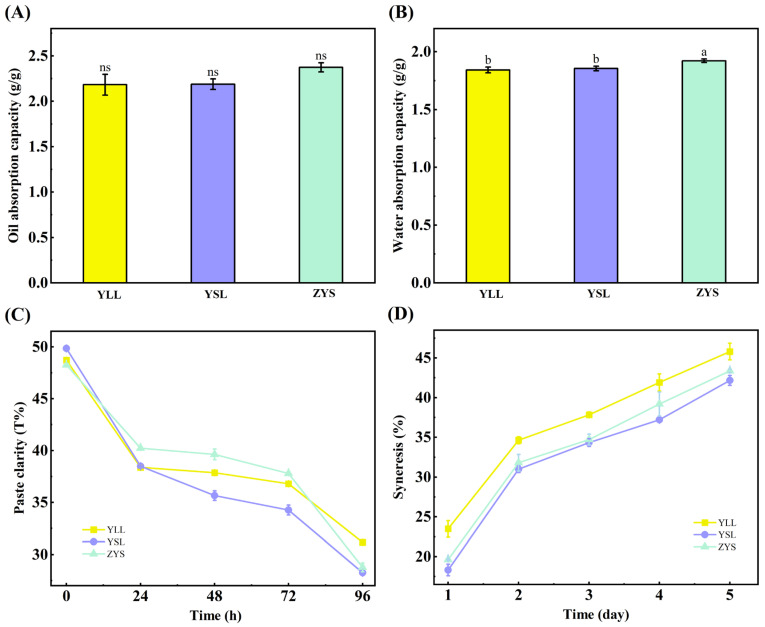
Oil absorption capacity (OAC), water absorption capacity (WAC), paste clarity, and syneresis of tiger nut starch. (**A**) OAC, (**B**) WAC, (**C**) paste clarity, (**D**) syneresis. Sample abbreviations: YLL, Yunnan Large-seeded Tiger Nut; YSL, Yunnan Small-seeded Tiger Nut; ZYS, Zhongyousha No.1. Values are presented as mean ± SD (*n* = 3). Different lowercase letters within the same group indicate significant differences. ns, not significant (one-way ANOVA followed by Duncan’s multiple range test, *p* < 0.05).

As shown in Figure 7A,B, significant differences in WAC were observed among the three tiger nut starches (*p* < 0.05), whereas inter-varietal variation in OAC was comparatively modest. ZYS exhibited the highest WAC (2.37 g/g) and a relatively high OAC (1.92 g/g). The elevated WAC of ZYS is consistent with its higher amylose content and smaller granule size—structural features that may respectively provide more accessible hydroxyl groups for water binding and greater surface areas for adsorption. The more modest inter-varietal variation in OAC suggests that oil absorption may be less sensitive to the specific structural differences among these varieties. The observed differences in WAC and OAC among varieties suggest that these structural attributes may influence the water- and oil-binding behavior of tiger nut starch, which could be relevant to their performance in food systems where water retention or fat binding is functionally important [53,54].

#### 3.2.3. Paste Clarity

Paste clarity is a key factor influencing the visual appeal of many starch-based food products [21,52]. As shown in Figure 7C, paste clarity varied significantly with both storage time and starch variety. At 0 h, YSL starch exhibited the highest transmittance (49.85%), followed by YLL (48.70%) and ZYS (48.25%). These results reflect inherent varietal differences in starch structure. Paste clarity values of 11–21% have been reported for loquat starches [22], while potato starches exhibit transmittance values ranging from 20% to 56% [50], indicating that tiger nut starches in this study possess relatively high paste clarity.

During refrigerated storage, all samples showed a progressive decline in transparency, a phenomenon consistent with previous reports on tiger nut starch [40] and attributable to increased turbidity arising from starch retrogradation [21,29]. The reassociation of polymer chains, particularly amylose, during cooling and refrigeration leads to cloudiness, aggregation, and phase separation [21]. Notably, ZYS displayed the greatest stability in paste clarity over time. This may be related to its higher amylose content, which could facilitate the formation of a more stable gel network that restricts the reassociation of amylopectin chains. Although YSL exhibited the highest initial transmittance, it underwent a more pronounced decline, which may reflect the combined influence of its lower amylose-to-amylopectin ratio, larger granule size, and other structural features [41]. The relatively high paste clarity and the varietal differences in storage stability observed among these starches may be relevant to their performance in food systems where visual appearance over time is a consideration.

#### 3.2.4. Pasting Properties

**Figure 8:**
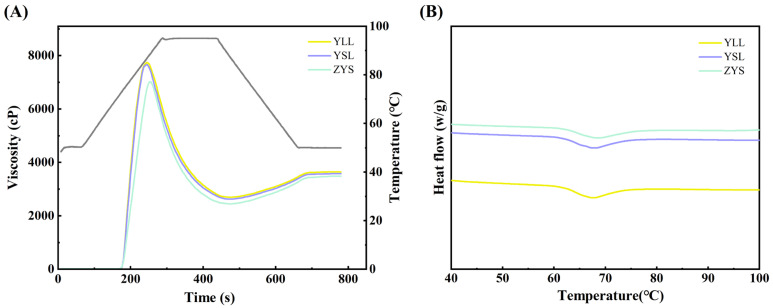
Pasting properties and thermal characteristics of tiger nut starch. (**A**) Rapid Visco Analyser (RVA) pasting curves, (**B**) Differential Scanning Calorimetry (DSC) thermograms. Sample abbreviations: YLL, Yunnan Large-seeded Tiger Nut; YSL, Yunnan Small-seeded Tiger Nut; ZYS, Zhongyousha No.1.

Gelatinization is an irreversible phase transition that occurs during starch heating, involving granule swelling, crystallite melting, and loss of birefringence [29]. The pasting properties of starch, which significantly influence the textural and sensory attributes of starch-based foods [55], varied significantly among the three tiger nut varieties (Figure 8A, Table 3). Pasting temperature (PT), an indicator of granule integrity, ranged narrowly from 72.80 to 73.22 °C with no significant differences among varieties, suggesting comparable initial swelling behavior. Peak viscosity (PV), which reflects swelling power, and final viscosity (FV), which indicates retrogradation tendency [56], differed markedly among the varieties. PV values ranged from 7030.0 to 7749.5 mPa·s, while FV values ranged from 3487.5 to 3648.0 mPa·s (*p* < 0.05). Higher PV tended to be observed in varieties with elevated amylopectin content, whereas higher FV was associated with a stronger retrogradation tendency.

Breakdown viscosity (BD), which assesses paste stability under high temperature and shear, was significantly lower for ZYS (4591.0 mPa·s) than for YSL (5045.5 mPa·s) and YLL (5057.0 mPa·s), suggesting that ZYS exhibited greater thermal and shear resistance [15]. Setback viscosity (SB), reflecting the propensity for amylose reassociation and short-term retrogradation during cooling [57], was also lowest for ZYS (1103.5 mPa·s) compared with YSL (1466.0 mPa·s) and YLL (1409.0 mPa·s), which may indicate reduced retrogradation and enhanced paste storage stability. The consistently lower PV, BD, and SB values observed for ZYS are consistent with its higher amylose content, smaller granule size, and higher relative crystallinity, suggesting that its distinct pasting behavior may be associated with this combination of structural features. These observations may serve as a preliminary reference for understanding the processing behavior of different tiger nut varieties.

#### 3.2.5. Rheological Properties

**Figure 9:**
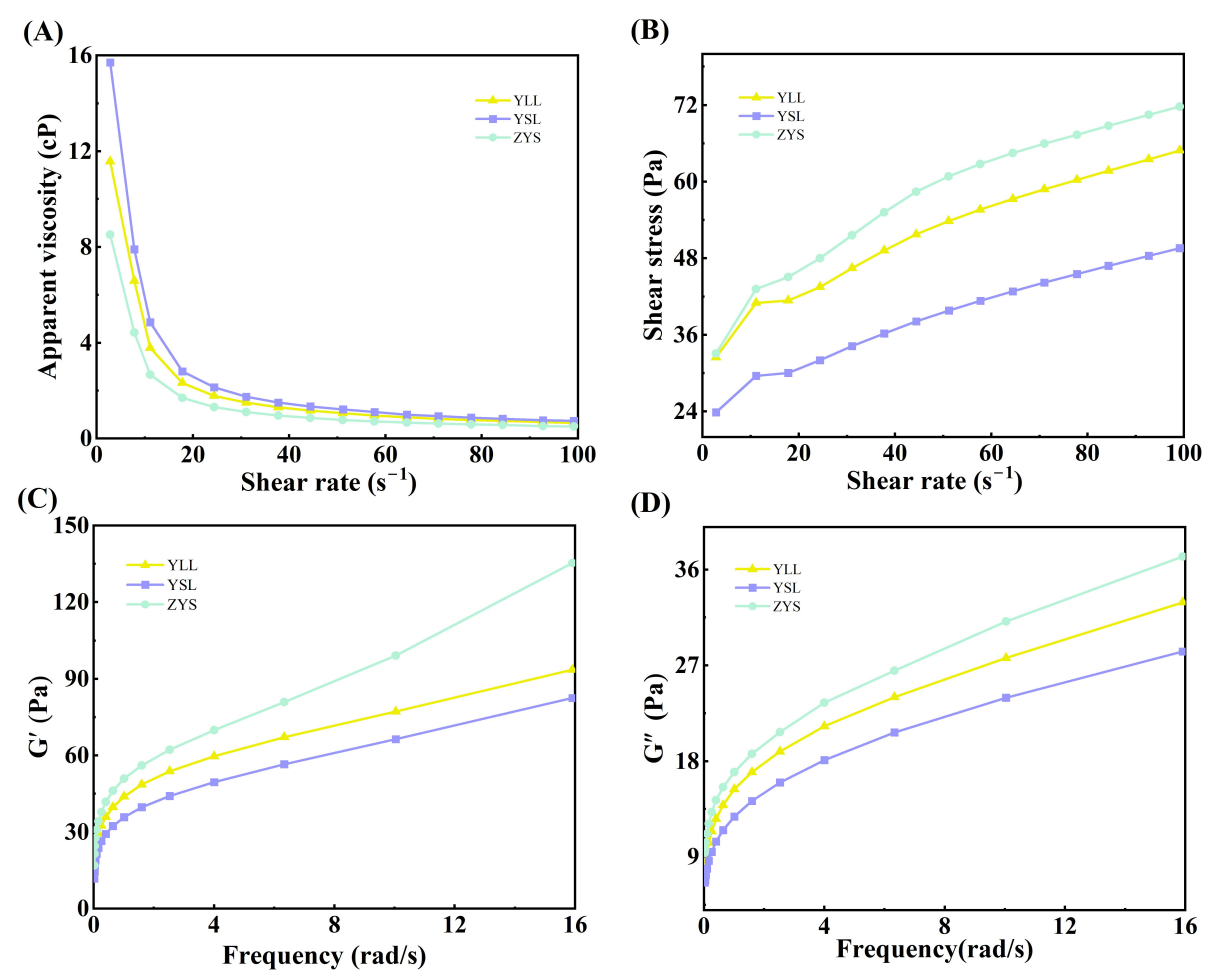
Rheological properties of tiger nut starch. (**A**) apparent viscosity, (**B**) shear rate, (**C**) storage modulus (G′), (**D**) loss modulus (G″). Sample abbreviations: YLL, Yunnan Large-seeded Tiger Nut; YSL, Yunnan Small-seeded Tiger Nut; ZYS, Zhongyousha No.1.

The rheological properties of the three tiger nut starches are shown in Table 4 and Figure 9. The shear stress–shear rate curves (Figure 9A,B) show that, for all starch samples, shear stress increased with shear rate, whereas apparent viscosity decreased rapidly at first and then leveled off. All samples exhibited a flow behavior index (*n*) below 1 (0.22–0.24), indicating non-Newtonian pseudoplastic behavior with pronounced shear-thinning in all three starches. This shear-thinning behavior is characteristic of pseudoplastic fluids and may offer practical advantages in processes such as pumping and mixing. Shear-thinning has been attributed to the breakdown of large starch granules, molecular chain scission, and subsequent reorientation [19]. Under shear, starch molecules gradually align with the flow direction, reducing intermolecular friction. Simultaneously, internal hydrogen and secondary bonds may break, causing the starch gel network to progressively degrade and apparent viscosity to decrease as shear rate increases. When the shear rate reaches a threshold, starch molecules become fully oriented or can no longer reorient, and the viscosity approaches a constant value [58]. According to the Ostwald–de Waele model, ZYS displayed a significantly lower consistency coefficient (*K*) compared with YLL and YSL. This suggests that ZYS starch possesses weaker thickening capacity. Furthermore, the steady-state apparent viscosity trends observed for all three starches were consistent with the final viscosity values obtained from RVA analysis.

Dynamic oscillatory measurements revealed that over the tested frequency range, the storage modulus (G′) exceeded the loss modulus (G″) for all three starches, with tan δ values remaining below 0.5 (Figure 9C,D). This indicates that the gelatinized systems were predominantly elastic in nature, exhibiting gel-like rheological behavior [40]. At a given frequency, ZYS displayed higher G′ and G″ values than YLL and YSL, suggesting a stronger elastic response under low-frequency conditions and the ability to retain some viscous character under high shear. The lower tan *δ* of ZYS suggests a relatively more elastic character of the gel network. The differences in both steady and dynamic rheological properties among the three tiger nut varieties appeared broadly consistent with their pasting behavior and structural characteristics, and were generally in line with previously reported findings on the rheological characteristics of tiger nut starch [15].

#### 3.2.6. Thermal Properties

The thermal properties of starch reflect the thermal stability of crystalline regions and the disruption of double-helical structures within starch granules [29]. The thermal characteristics of the three tiger nut starches are presented in Figure 8B and Table 5. All samples exhibited distinct endothermic peaks. The onset temperature (*To*) of YSL was the highest (62.26 °C), whereas ZYS displayed a lower value (61.62 °C). Peak temperatures (*Tp*) ranged from 67.40 to 68.16 °C, with YSL exhibiting a higher *Tp* than the other varieties. Conclusion temperatures (*Tc*) ranged from 77.29 to 78.70 °C, with YSL showing the highest value (78.70 °C) and YLL the lowest (77.29 °C). Gelatinization temperatures are associated with the stability of crystalline regions and granule size. The lower gelatinization temperatures observed for ZYS and YLL may be related to their smaller granule size and possibly to differences in the size distribution or organization of their crystalline domains. Although ZYS exhibited the highest relative crystallinity and the highest short-range molecular order, its gelatinization temperatures were not the highest among the three varieties, indicating that thermal stability is not solely determined by the quantity or quality of ordered structures. The ranking of gelatinization temperatures did not align with the ranking of crystallinity, suggesting that granule size and possibly other compositional variables collectively influence the thermal behavior of these starches.

Gelatinization enthalpy (Δ*H*) represents the energy required to disrupt the double-helical structure within starch crystalline domains [20]. Previous studies have demonstrated that Δ*H* is influenced by multiple factors, including the amylose-to-amylopectin ratio, granule size, and degree of crystallinity [29,52]. Δ*H* values of the three starches showed no significant differences, ranging from 12.67 to 13.49 J·g^−1^. YSL exhibited a slightly higher Δ*H* than ZYS and YLL, which may be related to its larger granule size—a structural feature that has been reported to contribute to greater double-helix stability during gelatinization [29,52]. The differing thermal properties among these varieties suggest that they may respond differently to thermal processing.

#### 3.2.7. Freeze–Thaw Stability

Freeze–thaw stability is a critical parameter for evaluating starch performance in frozen food systems and is commonly assessed by measuring syneresis (water release) after repeated freeze–thaw cycles, with higher syneresis indicating lower freeze–thaw stability [15]. As shown in Figure 7D, syneresis increased progressively with the number of freeze–thaw cycles for all three tiger nut starches, suggesting that repeated freezing and thawing promotes intermolecular associations among starch chains, thereby facilitating the release of bound water from the gel network. After five freeze–thaw cycles, syneresis reached 45.80% for YLL, 42.17% for YSL, and 43.37% for ZYS, in contrast to the corresponding values after the first cycle of 23.49%, 18.33%, and 19.65%, respectively Overall, YSL and ZYS exhibited lower syneresis than YLL, suggesting relatively better freeze–thaw stability. These varietal differences in freeze–thaw stability may be related to differences in amylose content, granule size, and retrogradation behavior among the varieties.

Following freeze–thaw treatment, starch gels typically develop a porous or sponge-like structure, generally attributed to mechanical damage inflicted on the gel network by ice crystal formation and growth, leading to partial network collapse and water expulsion [15]. Previous studies have reported that tiger nut starch exhibits syneresis values of approximately 45–58% after one to five freeze–thaw cycles, and was noted to display better freeze–thaw stability than corn, mung bean, and potato starches under the tested conditions [15], which is consistent with the present findings. The relatively low syneresis observed for YSL and ZYS suggests that these two varieties may maintain gel integrity better during freeze–thaw cycling, which could be an advantage in frozen food applications.

### 3.3. In Vitro Digestibility

The in vitro digestibility of the three tiger nut starches was systematically evaluated by quantifying rapidly digestible starch (RDS), slowly digestible starch (SDS), and resistant starch (RS) fractions [59]. RS escapes enzymatic hydrolysis in the small intestine and is fermented by colonic microbiota to produce short-chain fatty acids, conferring physiological benefits such as improved glycemic control, enhanced lipid metabolism, and promotion of gut health [43].

**Figure 10:**
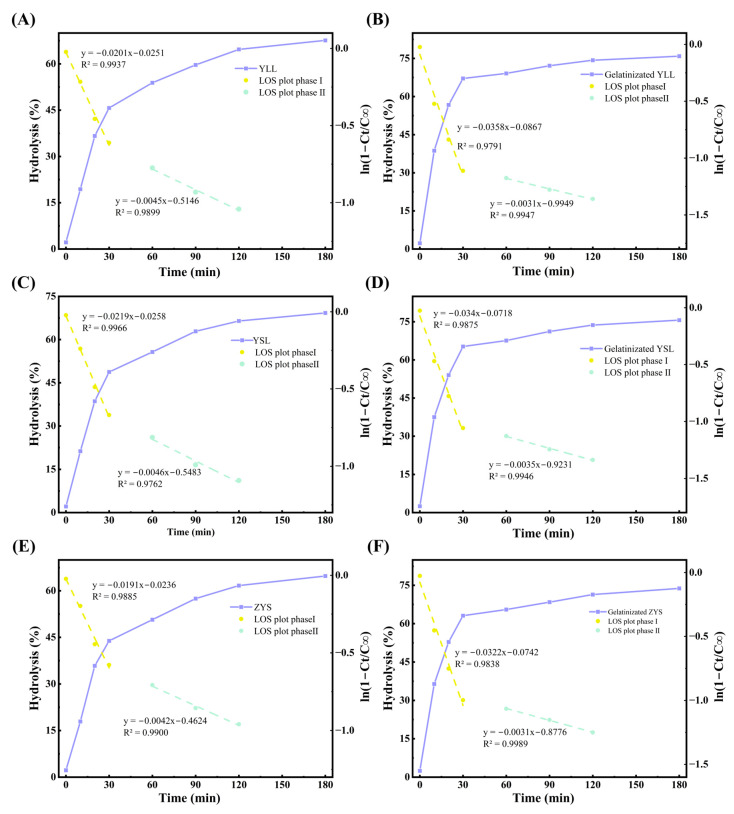
In vitro digestion of tiger nut starch. (**A**) In vitro digestion of YLL native starch, (**B**) In vitro digestion of YLL gelatinized starch, (**C**) In vitro digestion of YSL native starch, (**D**) In vitro digestion of YSL gelatinized starch, (**E**) In vitro digestion of ZYS native starch, (**F**) In vitro digestion of ZYS gelatinized starch. The corresponding Logarithm of Slope (LOS) plots for two digestion phases are displayed alongside each hydrolysis curve. Sample abbreviations: YLL, Yunnan Large-seeded Tiger Nut; YSL, Yunnan Small-seeded Tiger Nut; ZYS, Zhongyousha No.1.

The digestive characteristics of the three starches are summarized in Table 6 and Figure 10. All samples exhibited a distinct biphasic hydrolysis pattern comprising an initial rapid phase (Phase I, 0–30 min) followed by a slower phase (Phase II, 30–120 min). The digestion rate constants (*k*) for native starches in Phase I were 0.0201 (YLL), 0.0219 (YSL), and 0.0191 (ZYS); in Phase II, *k* values decreased markedly to 0.0045, 0.0046, and 0.0042, respectively. Following gelatinization, Phase I *k* increased substantially (to 0.0358, 0.0340, and 0.0322), whereas Phase II *k* remained largely unchanged. The biphasic LOS model provided a satisfactory fit for all samples, with the coefficient of determination (*R*^2^) ranging from 0.9762 to 0.9989. Compared with the single-phase model, the biphasic model consistently yielded higher *R*^2^ values and more homogeneous residual distributions, supporting the adequacy of the biphasic description of the hydrolysis kinetics. Based on the biphasic hydrolysis pattern observed and the low Phase I *k* values, the resistant starch in the native granules is predominantly of the RS2 type. This form of resistance arises from the dense crystalline structure and compact granule architecture, which limit enzyme diffusion and binding to the granule interior. The RDS, SDS, and RS contents were: YLL (34.46%, 28.05%, 35.30%), YSL (36.43%, 27.86%, 33.55%), and ZYS (33.24%, 25.83%, 38.31%), with significant differences among varieties (*p* < 0.05).

The observed differences in digestibility appeared to be related to multi-scale structural features among the varieties. During Phase I, the digestion rate was found to be associated with granule morphology, crystallinity, and amylose content. YSL exhibited the highest Phase I *k* value, which may be related to its lower crystallinity and relatively larger granule size. In contrast, ZYS, despite its smaller granules, displayed the lowest Phase I *k*, which may be related to its higher crystallinity that likely restricted enzyme accessibility. In Phase II, *k* values decreased substantially and inter-varietal differences were minimal, suggesting that digestion at this stage was probably constrained by the internal crystalline architecture once the outer granule layers had been hydrolyzed.

Structural attributes also appeared to be associated with differences in the RDS, SDS, and RS fractions. Among the three varieties, higher amylose content tended to be accompanied by greater RS content, whereas higher crystallinity and more ordered short-range molecular arrangement were linked to reduced enzyme accessibility and slower digestion [60,61]. These factors may collectively influence granule integrity, swelling capacity, and enzyme accessibility, thereby helping to explain, at least in part, to the variation in starch digestion rate. Gelatinization markedly altered the digestive characteristics: Phase I *k* values increased, RDS content rose, and both SDS and RS contents declined, consistent with heat-induced disruption of granular integrity and ordered domains, which enhances enzyme accessibility [43]. The gelatinized samples retained relatively high RS contents (22.70–25.64%), which may be related, at least in part, to the rapid reassociation of amylose chains (RS3 formation) upon cooling [62]. The RS contents of native tiger nut starches in this study appeared to fall within the upper range of values reported for common starches such as sweet potato, potato, cassava, wheat, and maize under similar experimental conditions [15,63], indicating that tiger nut starch may have potential as an ingredient for foods with reduced digestibility.

### 3.4. Structure–Property Relationships

The functional differences among the three varieties were consistent with the trends observed in their structural characteristics. ZYS and YSL can be regarded as representing two contrasting structural types, with YLL occupying an intermediate position between them. ZYS exhibited the highest amylose content (26.99%), the smallest granule size, and the highest relative crystallinity (30.60%), along with the highest short-range molecular order among the three varieties. YSL, in contrast, had the lowest amylose content (22.82%), the largest granules, and the lowest crystallinity (25.40%). YLL, with intermediate amylose content (25.91%) and crystallinity (29.47%), fell between the two extremes across most functional properties. Consistent with this structural gradient, ZYS displayed lower swelling power, lower peak viscosity, greater shear stability, and the highest resistant starch content (38.31%), whereas YSL showed the highest swelling power, the highest viscosity, and a faster digestion rate.

The structural basis for these functional differences can be understood from the interplay among amylose content, crystalline organization, and granule architecture. The high amylose content of ZYS, together with its high crystallinity, contributes to a dense molecular network within the granule that restricts hydration, limits granular swelling, and reduces enzyme accessibility [30]. These structural features are consistent with its low swelling power, low pasting viscosity, and high resistant starch content. Although ZYS also had the smallest granules—a feature that would generally favor greater water uptake—the restrictive effects of its high amylose content and crystallinity appeared to dominate its swelling and pasting behavior. Conversely, the low amylose content and low crystallinity of YSL, which were accompanied by larger granules, favored greater water uptake and more extensive swelling, leading to higher viscosity and faster enzymatic digestion.

Both long-range and short-range structural order were highest in ZYS: it showed the highest relative crystallinity and the highest R_1047/1022_ ratio among the three varieties. This indicates that, in ZYS, the crystalline domains are both abundant and well-ordered at the molecular scale. Although previous studies have shown that long-range and short-range order do not always vary in parallel [64,65], the convergence of these two independent measures in ZYS suggests that its crystalline structure is more fully developed across multiple structural scales compared with YSL and YLL. The higher crystallinity and more ordered short-range molecular arrangement of ZYS are consistent with its high resistant starch content, as both features contribute to a physical barrier that limits enzyme diffusion and binding to the granule interior [36]. In contrast, the lower crystallinity and lower short-range order of YSL would favor greater enzyme accessibility, consistent with its faster digestion rate and lower RS content. It should be acknowledged that FTIR ratios reflect short-range molecular order rather than crystallinity in the XRD sense, and their interpretation can be influenced by factors such as water content, amylose-to-amylopectin ratio, and mineral content. In the present study, all varieties were A-type starches measured under identical conditions, and the consistent trends between FTIR ratios and other structural parameters provide confidence in the reliability of these data.

While the contrasting structural profiles of ZYS and YSL can broadly account for their functional differences, the relative importance of individual structural features varied depending on the specific functional property under consideration. Thermal stability, as reflected by *T*_0_ and Δ*H*, appeared to be more closely related to granule size than to crystallinity or short-range order—YSL, with the largest granules, ranked highest in both parameters despite having the lowest crystallinity. Digestive resistance, by contrast, appeared to depend more on the abundance and continuity of ordered structures that restrict enzyme accessibility [36]—ZYS, with the highest crystallinity and short-range order, showed substantially higher RS content than YSL and YLL. These differing sensitivities may help to explain why the ranking of varieties was not uniform across all functional assays.

Taken together, the results of this study suggest that the functional and digestive properties of tiger nut starch reflect the combined influence of amylose content, granule size, and crystalline organization, rather than being determined by any single structural parameter. Among these features, amylose content and crystallinity appeared to be the primary determinants of swelling, pasting, and digestibility, while granule size exerted a more prominent influence on thermal stability. These associations are based on observations from three varieties and should be viewed as preliminary. Other compositional factors—such as residual proteins, non-starch polysaccharides, and mineral content—may also contribute to the functional behavior of these starches, and their potential role warrants further investigation.

## 4. Conclusions

This study compared the multi-scale structural characteristics, physicochemical properties, and in vitro digestibility of starches from three tiger nut varieties (YLL, YSL, and ZYS). Significant varietal differences were observed in amylose content, granule size distribution, relative crystallinity, and short-range molecular order. These structural variations were accompanied by differences in functional properties, including pasting behavior, thermal stability, swelling power, and freeze–thaw stability. Among the three varieties, ZYS and YSL represented two contrasting structural types, with ZYS exhibiting higher amylose content, higher crystallinity, and higher short-range molecular order—structural features that were associated with lower swelling power, greater shear stability, and higher resistant starch content. YSL showed the opposite profile, while YLL occupied an intermediate position.

The resistant starch (RS) content of the three varieties (33.55–38.31%) appeared to fall within the upper range of values reported for common starches, and higher RS content tended to be observed in varieties with higher amylose content and crystallinity. The findings indicate that the functional and digestive properties of tiger nut starch are associated with its multi-scale structural features in a variety-dependent manner, although the limited number of cultivars examined precludes definitive causal inferences. These results provide a reference for understanding the structure–property relationships of starches from different tiger nut varieties and may inform further research on this sustainable starch resource.

## Figures and Tables

**Table 1:** Characteristics, origins, and cultivation conditions of the three tiger nut varieties.

Samples	Region	Abbreviation	Characteristics	Growing Season (May–September)	Monthly Mean Temp.	Total Precipitation (May–September)	Mean Monthly Sunshine (May–September)	Soil Type
Yunnan Large-seeded Tiger Nut	Honghe, Yunnan, China	YLL	Slender, brownish-yellow skin	May–September	~22–24 °C	~1100–1200 mm	~200–220 h	Red soil, yellow soil
Yunnan Small-seeded Tiger Nut	Baoshan, Yunnan, China	YSL	Relatively long, brownish-yellow skin	May–September	~17–19 °C	~1100–1200 mm	~160–180 h	Red soil, paddy soil
Zhongyousha No.1	Tumxuk, Xinjiang, China	ZYS	Nearly round, brownish-black skin	May–September	~22–28 °C	~30–50 mm	~250–300 h	Brown desert soil, saline-alkali soil

**Table 2:** Granule size of tiger nut starch.

Samples	D_m_	D_10_	D_50_	D_90_
YLL	9.855 ± 0.006 ^a^	4.436 ± 0.055 ^b^	10.230 ± 0.01 ^a^	14.673 ± 0.015 ^b^
YSL	9.722 ± 0.026 ^b^	4.610 ± 0.021 ^a^	9.789 ± 0.004 ^b^	15.163 ± 0.074 ^a^
ZYS	8.129 ± 0.016 ^c^	1.759 ± 0.003 ^c^	8.661 ± 0.016 ^c^	12.370 ± 0.075 ^c^

Note: Data are expressed as mean ± SD (*n* = 3). Different lowercase letters in the same column indicate significant differences (one-way ANOVA followed by Duncan’s multiple range test, *p* < 0.05). Sample abbreviations: YLL, Yunnan Large-seeded Tiger Nut; YSL, Yunnan Small-seeded Tiger Nut; ZYS, Zhongyousha No.1. D_m_, volume-weighted mean diameter; D_10_, D_50_, and D_90_ represent the particle sizes at which 10%, 50%, and 90% of the total particle volume are smaller, respectively.

**Table 3:** The pasting characteristic parameters of tiger nut starch.

Samples	PV (mPa·s)	BD (mPa·s)	FV (mPa·s)	SB (mPa·s)	PT (°C)
YLL	7749.5 ± 85.56 ^a^	5057.0 ± 27.28 ^a^	3648.0 ± 15.57 ^a^	1409.0 ± 11.72 ^a^	73.22 ± 0.18 ^ns^
YSL	7670.5 ± 21.92 ^a^	5045.5 ± 28.99 ^a^	3579.5 ± 12.02 ^b^	1466.0 ± 16.97 ^a^	73.15 ± 0.21 ^ns^
ZYS	7030.0 ± 55.16 ^b^	4591.0 ± 52.33 ^b^	3487.5 ± 13.43 ^c^	1103.5 ± 38.89 ^b^	72.80 ± 0.36 ^ns^

Note: Data are expressed as mean ± SD (*n* = 3). Different lowercase letters in the same column indicate significant differences. ns, not significant (one-way ANOVA followed by Duncan’s multiple range test, *p* < 0.05). Sample abbreviations: YLL, Yunnan Large-seeded Tiger Nut; YSL, Yunnan Small-seeded Tiger Nut; ZYS, Zhongyousha No.1. PT stands for Pasting Temperature, PV for Peak Viscosity, BD for Breakdown Value, FV for Final Viscosity, and SB for Setback Value.

**Table 4:** Rheological parameters of tiger nut starch.

Samples	*K* (Pa∙s^n^)	*n*	R^2^	tan *δ* (10 s^−1^)
YLL	23.53 ± 0.53 ^a^	0.2357 ± 0.47 ^ns^	0.9871	0.36 ± 0.03 ^a^
YSL	22.86 ± 0.83 ^a^	0.2251 ± 0.79 ^ns^	0.9653	0.36 ± 0.05 ^a^
ZYS	15.61 ± 0.54 ^b^	0.2334 ± 0.51 ^ns^	0.9582	0.31 ± 0.03 ^b^

Note: Data are expressed as mean ± SD (*n* = 3). Different lowercase letters in the same column indicate significant differences. ns, not significant (one-way ANOVA followed by Duncan’s multiple range test, *p* < 0.05). Sample abbreviations: YLL, Yunnan Large-seeded Tiger Nut; YSL, Yunnan Small-seeded Tiger Nut; ZYS, Zhongyousha No.1. *K* represents consistency coefficient, *n* represents flow behavior index, and tan *δ* represents the ratio of storage modulus to loss modulus.

**Table 5:** The thermal characteristic parameters of tiger nut starch.

Samples	*To* (°C)	*Tp* (°C)	*Tc* (°C)	Δ*H* (J/g)
YLL	61.97 ± 0.13 ^a^	67.40 ± 0.07 ^b^	77.29 ± 0.41 ^b^	12.67 ± 0.08 ^ns^
YSL	62.26 ± 0.06 ^a^	68.16 ± 0.32 ^a^	78.70 ± 0.10 ^a^	13.49 ± 0.71 ^ns^
ZYS	61.62 ± 0.22 ^b^	67.49 ± 0.24 ^b^	77.62 ± 0.05 ^b^	12.86 ± 0.02 ^ns^

Note: Data are expressed as mean ± SD (*n* = 3). Different lowercase letters in the same column indicate significant differences. ns, not significant (one-way ANOVA followed by Duncan’s multiple range test, *p* < 0.05). Sample abbreviations: YLL, Yunnan Large-seeded Tiger Nut; YSL, Yunnan Small-seeded Tiger Nut; ZYS, Zhongyousha No.1. *To* represents onset temperature, *Tp* represents peak temperature, *Tc* represents conclusion temperature, and Δ*H* represents gelatinization enthalpy.

**Table 6:** In vitro digestibility characteristics of gelatinized and ungelatinized tiger nut starch.

Samples	Ungelatinized			Gelatinized		
	RDS	SDS	RS	RDS	SDS	RS
YLL	34.46 ± 0.15 ^b^	28.05 ± 0.90 ^ns^	35.30 ± 0.90 ^b^	54.64 ± 0.31 ^a^	21.76 ± 1.59 ^ns^	22.70 ± 0.08 ^b^
YSL	36.43 ± 0.49 ^a^	27.86 ± 0.08 ^ns^	33.55 ± 0.08 ^b^	51.51 ± 0.73 ^ab^	22.78 ± 0.11 ^ns^	22.28 ± 0.23 ^b^
ZYS	33.24 ± 0.56 ^b^	25.83 ± 0.21 ^ns^	38.31 ± 0.21 ^a^	50.37 ± 0.34 ^b^	22.64 ± 0.24 ^ns^	25.64 ± 0.11 ^a^

Note: Data are expressed as mean ± SD (*n* = 3). Different lowercase letters in the same column indicate significant differences. ns, not significant (one-way ANOVA followed by Duncan’s multiple range test, *p* < 0.05). Sample abbreviations: YLL, Yunnan Large-seeded Tiger Nut; YSL, Yunnan Small-seeded Tiger Nut; ZYS, Zhongyousha No.1. RDS, rapidly digestible starch; SDS, slowly digestible starch; RS, resistant starch.

## Data Availability

The original contributions presented in this study are included in the article. Further inquiries can be directed to the corresponding author.
